# Supplementary material for: Iterative improvement in the automatic modular design of robot swarms
Source: PeerJ Comput Sci. 2020 Dec 7;6:e322. doi: 10.7717/peerj-cs.322 (PMC7924708; doi:10.7717/peerj-cs.322)
Supplement: Supplemental Information 3 [file peerj-cs-06-322-s003.zip › argos3/doc/api/standalone/a00613_source.html]

ARGoS: plugins/simulator/physics\_engines/dynamics2d/dynamics2d\_single\_body\_object\_model.cpp Source File


- Main Page
- Related Pages
- Namespaces
- Classes
- Files

- File List
- File Members

# plugins/simulator/physics\_engines/dynamics2d/dynamics2d\_single\_body\_object\_model.cpp

Go to the documentation of this file.

```
00001 #include "dynamics2d_single_body_object_model.h"
00002 #include <argos3/core/simulator/entity/composable_entity.h>
00003 
00004 namespace argos {
00005 
00006    /****************************************/
00007    /****************************************/
00008 
00009    CDynamics2DSingleBodyObjectModel::CDynamics2DSingleBodyObjectModel(CDynamics2DEngine& c_engine,
00010                                                                       CComposableEntity& c_entity) :
00011       CDynamics2DModel(c_engine, c_entity.GetComponent<CEmbodiedEntity>("body")),
00012       m_cEntity(c_entity),
00013       m_ptBody(NULL) {}
00014 
00015    /****************************************/
00016    /****************************************/
00017 
00018    CDynamics2DSingleBodyObjectModel::~CDynamics2DSingleBodyObjectModel() {
00019       bool bIsStatic = cpBodyIsStatic(m_ptBody);
00020       /* Dispose of shapes */
00021       cpShape* ptCurShape = m_ptBody->shapeList;
00022       cpShape* ptNextShape;
00023       while(ptCurShape) {
00024          ptNextShape = ptCurShape->next;
00025          cpSpaceRemoveShape(GetDynamics2DEngine().GetPhysicsSpace(), ptCurShape);
00026          cpShapeFree(ptCurShape);
00027          ptCurShape = ptNextShape;
00028       }
00029       /* Dispose of body */
00030       if(! bIsStatic)
00031          cpSpaceRemoveBody(GetDynamics2DEngine().GetPhysicsSpace(), m_ptBody);
00032       cpBodyFree(m_ptBody);
00033       /* Reindex space */
00034       if(bIsStatic) cpSpaceReindexStatic(GetDynamics2DEngine().GetPhysicsSpace());
00035    }
00036 
00037    /****************************************/
00038    /****************************************/
00039 
00040    void CDynamics2DSingleBodyObjectModel::MoveTo(const CVector3& c_position,
00041                                                  const CQuaternion& c_orientation) {
00042       /* Move the body to the desired position */
00043       m_ptBody->p = cpv(c_position.GetX(), c_position.GetY());
00044       CRadians cXAngle, cYAngle, cZAngle;
00045       c_orientation.ToEulerAngles(cZAngle, cYAngle, cXAngle);
00046       cpBodySetAngle(m_ptBody, cZAngle.GetValue());
00047       /* Update shape index */
00048       if(cpBodyIsStatic(m_ptBody)) {
00049          cpBB tBoundingBox = cpShapeGetBB(m_ptBody->shapeList);
00050          cpSpaceReindexStatic(GetDynamics2DEngine().GetPhysicsSpace());
00051          tBoundingBox = cpShapeGetBB(m_ptBody->shapeList);
00052       }
00053       else {
00054          cpSpaceReindexShapesForBody(GetDynamics2DEngine().GetPhysicsSpace(), m_ptBody);
00055       }
00056       /* Update ARGoS entity state */
00057       CDynamics2DModel::UpdateEntityStatus();
00058    }
00059 
00060    /****************************************/
00061    /****************************************/
00062 
00063    void CDynamics2DSingleBodyObjectModel::Reset() {
00064       /* Nothing to do for a static body */
00065       if(cpBodyIsStatic(m_ptBody)) return;
00066       /* Reset body position */
00067       const CVector3& cPosition = GetEmbodiedEntity().GetOriginAnchor().Position;
00068       m_ptBody->p = cpv(cPosition.GetX(), cPosition.GetY());
00069       /* Reset body orientation */
00070       CRadians cXAngle, cYAngle, cZAngle;
00071       GetEmbodiedEntity().GetOriginAnchor().Orientation.ToEulerAngles(cZAngle, cYAngle, cXAngle);
00072       cpBodySetAngle(m_ptBody, cZAngle.GetValue());
00073       /* Zero speed and applied forces */
00074       m_ptBody->v = cpvzero;
00075       m_ptBody->w = 0.0f;
00076       cpBodyResetForces(m_ptBody);
00077       /* Update bounding box */
00078       cpSpaceReindexShapesForBody(GetDynamics2DEngine().GetPhysicsSpace(), m_ptBody);
00079       CalculateBoundingBox();
00080    }
00081 
00082    /****************************************/
00083    /****************************************/
00084 
00085    void CDynamics2DSingleBodyObjectModel::CalculateBoundingBox() {
00086       cpBB tBoundingBox = cpShapeGetBB(m_ptBody->shapeList);
00087       for(cpShape* pt_shape = m_ptBody->shapeList->next;
00088           pt_shape != NULL;
00089           pt_shape = pt_shape->next) {
00090          cpBB* ptBB = &pt_shape->bb;
00091          if(ptBB->l < tBoundingBox.l) tBoundingBox.l = ptBB->l;
00092          if(ptBB->b < tBoundingBox.b) tBoundingBox.b = ptBB->b;
00093          if(ptBB->r > tBoundingBox.r) tBoundingBox.r = ptBB->r;
00094          if(ptBB->t > tBoundingBox.t) tBoundingBox.t = ptBB->t;
00095       }
00096       GetBoundingBox().MinCorner.SetX(tBoundingBox.l);
00097       GetBoundingBox().MinCorner.SetY(tBoundingBox.b);
00098       GetBoundingBox().MaxCorner.SetX(tBoundingBox.r);
00099       GetBoundingBox().MaxCorner.SetY(tBoundingBox.t);
00100    }
00101 
00102    /****************************************/
00103    /****************************************/
00104 
00105    void CDynamics2DSingleBodyObjectModel::UpdateEntityStatus() {
00106       /* Nothing to do for a static body */
00107       if(!cpBodyIsStatic(m_ptBody)) {
00108          CDynamics2DModel::UpdateEntityStatus();
00109       }
00110    }
00111 
00112    /****************************************/
00113    /****************************************/
00114 
00115    bool CDynamics2DSingleBodyObjectModel::IsCollidingWithSomething() const {
00116       for(cpShape* pt_shape = m_ptBody->shapeList;
00117           pt_shape != NULL;
00118           pt_shape = pt_shape->next) {
00119          if(cpSpaceShapeQuery(
00120                const_cast<CDynamics2DSingleBodyObjectModel*>(this)->
00121                GetDynamics2DEngine().GetPhysicsSpace(),
00122                pt_shape, NULL, NULL) > 0) {
00123             return true;
00124          }
00125       }
00126       return false;
00127    }
00128 
00129    /****************************************/
00130    /****************************************/
00131 
00132    void CDynamics2DSingleBodyObjectModel::SetBody(cpBody* pt_body,
00133                                                   Real f_height) {
00134       /* Set the body and its data field for ray queries */
00135       m_ptBody = pt_body;
00136       m_ptBody->data = this;
00137       /* Register the origin anchor update method */
00138       RegisterAnchorMethod(GetEmbodiedEntity().GetOriginAnchor(),
00139                            &CDynamics2DSingleBodyObjectModel::UpdateOriginAnchor);
00140       /* Calculate the bounding box */
00141       GetBoundingBox().MinCorner.SetZ(GetEmbodiedEntity().GetOriginAnchor().Position.GetZ());
00142       GetBoundingBox().MaxCorner.SetZ(GetEmbodiedEntity().GetOriginAnchor().Position.GetZ() + f_height);
00143       CalculateBoundingBox();
00144    }
00145 
00146    /****************************************/
00147    /****************************************/
00148 
00149    void CDynamics2DSingleBodyObjectModel::UpdateOriginAnchor(SAnchor& s_anchor) {
00150       s_anchor.Position.SetX(m_ptBody->p.x);
00151       s_anchor.Position.SetY(m_ptBody->p.y);
00152       s_anchor.Orientation.FromAngleAxis(CRadians(m_ptBody->a), CVector3::Z);
00153    }
00154 
00155    /****************************************/
00156    /****************************************/
00157 
00158 }
```

---

Generated on 10 Jul 2018 for ARGoS by 
 1.6.1
